# Deciphering the role of IL17RA in psoriasis and chronic mucocutaneous candidiasis: shared pathways and distinct manifestations

**DOI:** 10.3389/fimmu.2024.1516408

**Published:** 2025-01-20

**Authors:** Ayat Kadhi, Edward Eid, Michel J. Massaad, Inaam El-Rassy, Dana Maria Khoury, Yutaka Shimomura, Nelly Rubeiz, Mazen Kurban, Georges Nemer

**Affiliations:** ^1^ College of Health and Life Sciences, Hamad Bin Khalifa University, Doha, Qatar; ^2^ College of Health and Sciences, University of Doha for Science and Technology, Doha, Qatar; ^3^ Human Genetics Department, Sidra Medicine, Doha, Qatar; ^4^ Department of Dermatology, Faculty of Medicine, American University of Beirut, Beirut, Lebanon; ^5^ Department of Experimental Pathology, Immunology, and Microbiology, Faculty of Medicine, American University of Beirut, Beirut, Lebanon; ^6^ Pillar Genomic Institute (PGI), Faculty of Medicine, American University of Beirut, Beirut, Lebanon; ^7^ Department of Dermatology, Yamaguchi University Graduate School of Medicine, Ube, Japan; ^8^ Department of Biochemistry and Molecular Genetics, Faculty of Medicine, American University of Beirut, Beirut, Lebanon

**Keywords:** IL17RA, psoriasis, oral candidiasis, multi-omics, flow cytometry

## Abstract

**Introduction:**

Psoriasis and chronic mucocutaneous candidiasis (CMC), although distinct in their clinical manifestations, often coexist within specific patient cohorts. Despite this intriguing clinical observation, their genetic etiologies have been studied separately, neglecting the shared inflammatory mediator, interleukin 17A-F (IL17A-F). Consequently, the immunogenetic foundations underlying these conditions have remained enigmatic.

**Methods:**

In this study, we analyzed the case of a 5-year-old female born to consanguineous parents who presented with concomitant psoriasis and CMC phenotypes. Utilizing whole exome and transcriptomic sequencing, we meticulously investigated the genetic underpinnings and molecular pathways underlying these complex pathologies. RNA sequencing was performed on a skin biopsy to confirm transcriptomic profiles associated with these conditions.

**Results:**

We identified a novel bi-allelic variant (NM_014339.6, c.1173C>G A) within the interleukin 17 receptor type A (IL17RA) gene, resulting in a premature stop codon (p. Tyr391Ter). Despite the truncation, our investigations revealed that this variant produces a fully functional IL17RA protein. This was evident from the presence of IL17RA in the patient’s peripheral blood mononuclear cells (PBMCs) and the ability of the mutant IL17RA to dimerize with both wild-type protein and its partners IL17RC and IL17RD. Transcriptomic analysis of the skin biopsy showed a distinct psoriasis-associated signature intertwined with inflammatory pathways, including responses to fungal infections.

**Discussion:**

This report unveils an unprecedented genetic link serving as a common denominator for psoriasis and CMC. The novel IL17RA variant highlights the pivotal role of this receptor in the shared inflammatory pathways underlying these conditions. Our findings bridge a critical knowledge gap and provide insights into the molecular mechanisms connecting these diseases. This discovery not only advances our understanding of their pathophysiology but also lays the groundwork for personalized therapeutic strategies, heralding a new era of precision medicine for patients with intertwined psoriasis and CMC.

## Graphical Abstract

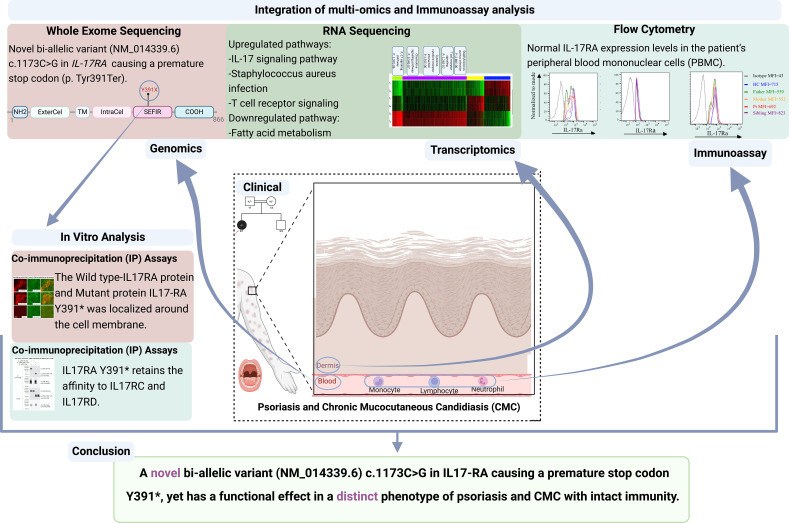

## Highlights

- Novel pathogenic biallelic nonsense variant (NM_014339.6) c.1173C>G identified in the *IL17RA* gene by Whole Exome Sequencing in a 5-year-old child with psoriasis and chronic mucocutaneous candidiasis, expanding the range of IL17RA variant-associated phenotypes.
- The identified variant (NM_014339.6) c.1173C>G results in a premature stop codon with an amino acid change (p. Tyr391Ter). Despite that, the variant retains its functionality; it is expressed around the cell membrane, interacts with IL17RC and IL17RD partners like the wild type, and shows similar expression of IL17RA in peripheral blood mononuclear cells (PBMC) between the affected child and non-affected family members.
- Transcriptome analysis reveals signature pathways linked to psoriasis, and candidiasis, with IL17 identified as an upstream regulator.
- IL17/IL17RA axis SNPs potentially contribute to variable responses to IL17 inhibitors, warranting further investigation.

## Introduction

Psoriasis and chronic mucocutaneous candidiasis (CMC) represent distinct yet interconnected inflammatory conditions, exerting a profound impact on affected individuals’ quality of life. Chronic mucocutaneous candidiasis (OMIM#615527, CMC) is a rare immunodeficiency disorder, characterized by recurrent and persistent fungal infections affecting the oral mucosa primarily (1). Psoriasis (OMIM: 614204), a chronic inflammatory skin disorder, is characterized by the presence of red plaques anywhere in the body, excessive keratinocyte proliferation, and immune cell infiltration driven by dysregulated T helper 17 (Th17), which are defined by their production of Interleukin-17 (IL-17) in response to Interleukin-23 (IL-23) (2). IL-23 promotes the survival and proliferation of Th17 cells, which in turn secrete pro-inflammatory cytokines such as IL-17A and IL-22. These cytokines contribute to the inflammatory response and keratinocyte proliferation seen in psoriatic lesions (3).

Interestingly, psoriasis exacerbations have been associated with *Candida albicans* infections, as individuals with psoriasis are shown to have higher rates of *Candida* colonization, particularly in saliva and sometimes on the skin and in feces, often without a clear etiology (4–7).

The interleukin-17 axis serves as a crucial defense mechanism against *Candida* albicans, while simultaneously playing a pivotal role in psoriasis immunopathogenesis (8, 9).

The role of Interleukin receptor A (IL17RA), a critical component of the IL17 receptor family is particularly significant, as genetic mutations in *IL17RA* have been linked to CMC (8, 10, 11). On the other hand, the association between IL17RA and psoriasis has been explored through association studies only, identifying a single nucleotide polymorphism (SNP) in *IL17RA* (rs4819554) that increases psoriasis risk in Spanish and Egyptian populations (9, 12).

Thus, IL17 inhibitors, often hailed as a breakthrough in the treatment of psoriasis for their precision in targeting the IL17 pathway come with a caveat.

Inhibiting the IL17 response may inadvertently promote fungal colonization, potentially aggravating the IL17 response and exacerbating psoriasis in a vicious cycle. This is exemplified by the common occurrence of oral candidiasis as a significant side effect of IL17 inhibitors (7). Of note, Brodalumab, the only approved anti-IL17RA drug for psoriasis, demonstrated superiority over ustekinumab in achieving Psoriasis Area Severity Index (PASI) 100 at week 12 in AMAGINE-2 and AMAGINE-3 clinical trials and a multicenter study involving 606 patients further showed that 91.3% of patients achieved a PASI score of 2 or less after 3 years of Brodalumab treatment (13, 14). However, the long-term use of Brodalumab was associated with a significantly higher rate of Candida infections compared to ustekinumab and other IL-17 inhibitors (13, 14).

Indeed, it is plausible that genetic mutations in the *IL17RA* gene could exacerbate this effect, an area that remains unexplored to our knowledge.

This study marks an unprecedented advancement in the understanding of these conditions by reporting the first mutation in *IL17RA* bridging psoriasis and CMC immunogenetic elements. Through comprehensive analysis, we aim to unravel the intricate immunogenetic underpinnings shared by these two conditions and potentially serve as a foundational cornerstone for deciphering the complex interactions shaping psoriasis and CMC pathogenesis and potentially pave the way for novel therapeutic strategies.

## Materials and methods

### Patients’ recruitment and DNA samples

Patients and their families were recruited at the Genodermatoses unit, department of dermatology at the American University of Beirut Medical Center (AUBMC, Beirut, Lebanon) under the ethical approval Institutional Review Board (IRB) from AUBMC (Protocol Number: IRB: DER.MK.01). Approval and written informed consents were obtained from patients or their parents (if minors) for obtaining blood sample and pictures. The sibling did not participate, and his genetic data was not available. Clinical phenotypes were provided by the referring physician. Peripheral blood samples were collected from the participating individuals and kept at 4 degrees Celsius. DNA was extracted within 1 hour after blood collection from specimens and stored at 4°C.

### Whole exome sequencing, annotation, and filter

Whole Exome Sequencing was performed for the patient and her parents at Macrogen Laboratory (https://dna.macrogen.com/)(Seoul, South Korea). 4 μg DNA of each sample was processed. One-hundred fifty-one base pair(bp-paired-end) reads were sequenced using the Illumina NovaSeq6000 platform. The library preparation was performed according to the manufacturer’s protocol (Twist Human core exome). The Phred quality value was assigned to Q30, which means that the base call accuracy was 99.9%. **Supplementary Table S1** shows the raw data statistics and quality scores. The generated FASTQ files were mapped to the Human GRCh37/hg19 reference assembly. Annotation and filtering processes for curated potential variants were performed as previously described (15).

### RNA extraction and sequencing

Punch biopsies were obtained from the lesion of the affected patient (MK384) and controls (MK391 and MK392), individuals without skin autoimmune or chronic inflammatory diseases who are around the same age as the patient. Samples were processed directly for total RNA extraction using the Invitrogen Life Technologies TRIzol reagent following the manufacturer’s protocol. RNA quantification was performed using the Nanodrop 1000 spectrophotometer (Thermo Scientific) at AUB. All samples, from both the patient and controls, were processed in technical duplicates, and the fragments per kilobase of exon per million mapped fragments (FKPM) values represent the mean of these duplicates. Further experimental details were performed as previously described (16).

### Differential expression analysis

Htseq-count was used to summarize the read counts at the gene level. The differentially expressed genes (DEGs) were identified using DESeq2 after filtering out genes with low counts in all samples from the expression matrix. A gene was considered differentially expressed if its adjusted P-value (false discovery rate or FDR) was less than 0.01, and the absolute Log2 fold change was greater than or equal to 1, which corresponds to a 2-fold change threshold. Differential expression analyses compared the gene-expression profiles between the patient and the controls. Data analysis was conducted using iDEP.91 for generating heatmaps, scatterplots, and boxplots. Hierarchical clustering with the top 1000 most variable genes used a correlationbased distance metric, with linkage determined by an average cut-off score of 4. Gene expression values were centered by subtracting their mean value. Additionally, k-mean clustering with four clusters was performed using the top 2000 variable genes to identify biological processes. Gene normalization was achieved using the mean-center pathway database for GO biological processes within iDEP. 91.

### Functional annotation analysis

The lists of DEGs were imported to the Ingenuity Pathway Analysis (IPA) software package (Ingenuity Systems, Redwood City, CA) to identify top enriched canonical pathways, upstream regulators, mechanistic networks, disease and functions’ annotations. The analysis followed a methodology similar to the description reported by (17). In brief, the probe sets were mapped to the Ensemble and HUGO gene symbols within IPA software. Probe sets that did not map to any HUGO/Ensemble genes were discarded. Pathways were ranked according to p-values, with the most significantly impacted pathways displayed at the top. The analysis included genes with log2 fold changes of < -4.4 and >+ 4.7 and FDR less than or equal to 0.05. These criteria were used to identify genes with the most pronounced differential expression, which were subsequently selected for functional annotation.

### Flow cytometry

Peripheral blood mononuclear cells (PBMCs) were isolated using a Ficoll-Paque PLUS gradient (GE Healthcare; IL, USA). Anti-human antibodies for surface makers, with isotype-matched controls, were used to stain CD3 (UCHT1) and CD14 (M5E2) from Biolegend (CA, USA), and IL-17RA (W23-251) from BD Biosciences (NJ, USA). In brief, T cells were identified by gating on the lymphocytes in the forward vs. side scatter plots using the CD3 marker, a pan marker for T lymphocytes. Monocytes were identified by gating on the monocytes in the forward vs. side scatter and using the CD14 marker, which is expressed on all monocytes and is part of the TLR4 signaling complex. Neutrophils were not specifically gated using a marker; instead, granulocytes, primarily composed of neutrophils, were gated in the forward vs. side scatter plots. Cells were sorted using a BD FACS Aria cell sorter (BD Biosciences, NJ, USA), and analyzed with FlowJo software (Tree Star Inc.; OR, USA).

### Generation of expression vectors

The vectors were generated at Yamaguchi University Graduate School of Medicine, Japan by Dr. Yutaka Shimomura. Tags were introduced into the C-terminus. Using skin cDNA from a healthy control individual, expression vectors were constructed for an N-terminal HA-tagged IL17RA (IL17RA-HA), IL17RA-Y391*-HA, and Il17RC-FLag. For wild-type (Wt) IL17RA, the coding sequences were amplified by polymerase chain reaction (PCR) using a forward primer (IL17RA-F-EcoRI: 5’-CTCTGAATTCTCAGAACGTTCGTTCGCTGC-3’) and a reverse primer (IL17RA-Wt-R-HA-SalI: 5’AAAAGTCGACTAGGCGTAGTCGGGCACGTCGTAGGGGTATGCACTGGGCCCCTCTGACT-3’) which introduced the HA-tag. The amplified PCR product was digested with EcoRI & SalI enzymes and subcloned into the pCXN2.1 vector at EcoRI and XhoI sites.

For the IL17RA-Y391*-HA vector, PCR was performed using the forward primer to amplify its sequence (IL17RA-F-EcoRI: 5’-CTCTGAATTCTCAGAACGTTCGTTCGCTGC-3’) and reverse primer (IL17RA-Y391*-R-HA-KpnI: 5’AAAGGTACCTAGGCGTAGTCGGGCACGTCGTAGGGGTAGAGGGGGTGGTCGGCT-3’) that introduced the reverse primer. The amplified product was digested with EcoRI and KpnI enzymes and subcloned into the mammalian pCXN2.1 vector at EcoRI and KpnI sites. The mutation c.1173C > G (p. Tyr391Ter) was introduced into the pCXN2.1- HA-IL17RA-Wt vector using the Quick-Change site-directed mutagenesis kit (Agilent Technologies). Similar to the cloning of IL17RA, cDNA sequences encoding the intracellular domain (IC) of IL17RC and IL17RD were amplified by PCR using forward and reverse primers. For IL17RC-Flag, the forward primer (IL17RC-F-EcoRI:5’CTCGAATTCGCCACCATGCCTGTGCCCTGGTTCTT-3’) and reverse primer (IL17RC-R-Flag-NheI): 5’AAAGCTAGCTACTTATCGTCGTCATCCTTGTAATCAGTCCCGTCCCCCGCCCCA-3’) were used. The HA-tag nucleotide sequences were introduced into the reverse primer. For all tags, it was introduced into the C-terminus. The PCR products were digested with EcoRI and NheI enzymes and subcloned into pCXN2.1 vector at EcoRI and NheI sites (pCXN2.1-IL17RC/D-HA).

### Cell culture, indirect immunofluorescence, co-immunoprecipitation assays

Human embryonic kidney (HEK293T) cells were used according to the manufacturer’s instructions. 0.6 μg of vectors encoding N-terminal HA-tagged IL17RA (Wt or p. Tyr391Termutant [Mut]) or an empty pCXN2.1 vector, were transfected into wells.

For immunofluorescence (IIF) studies, HEK293T cells were transfected with expression vectors for IL17RA-Wt-HA, IL17RA-Y391*-HA, or an empty vector (50 ng each). Cells were stained with rabbit polyclonal anti-HA antibody (diluted 1:1,000; Abcam) and mouse monoclonal antipan-cadherin antibody (diluted 1:1,000; Abcam). For Co-IP assays, HEK293T cells were transfected with IL17RA-HA and IL17RC-Flag, as well as IL17RA-HA and IL17RD-Flag (1.0 μg each), using Lipofectamine 2000 (Life Technologies). Immunoprecipitation was performed using mouse anti-DDDDK (flag) agarose gels (MBL International). Cell culture, transfection, IIF, and Co-IP were performed as previously described (16).

## Results

### Clinical diagnosis of two traits

**Figure 1:**
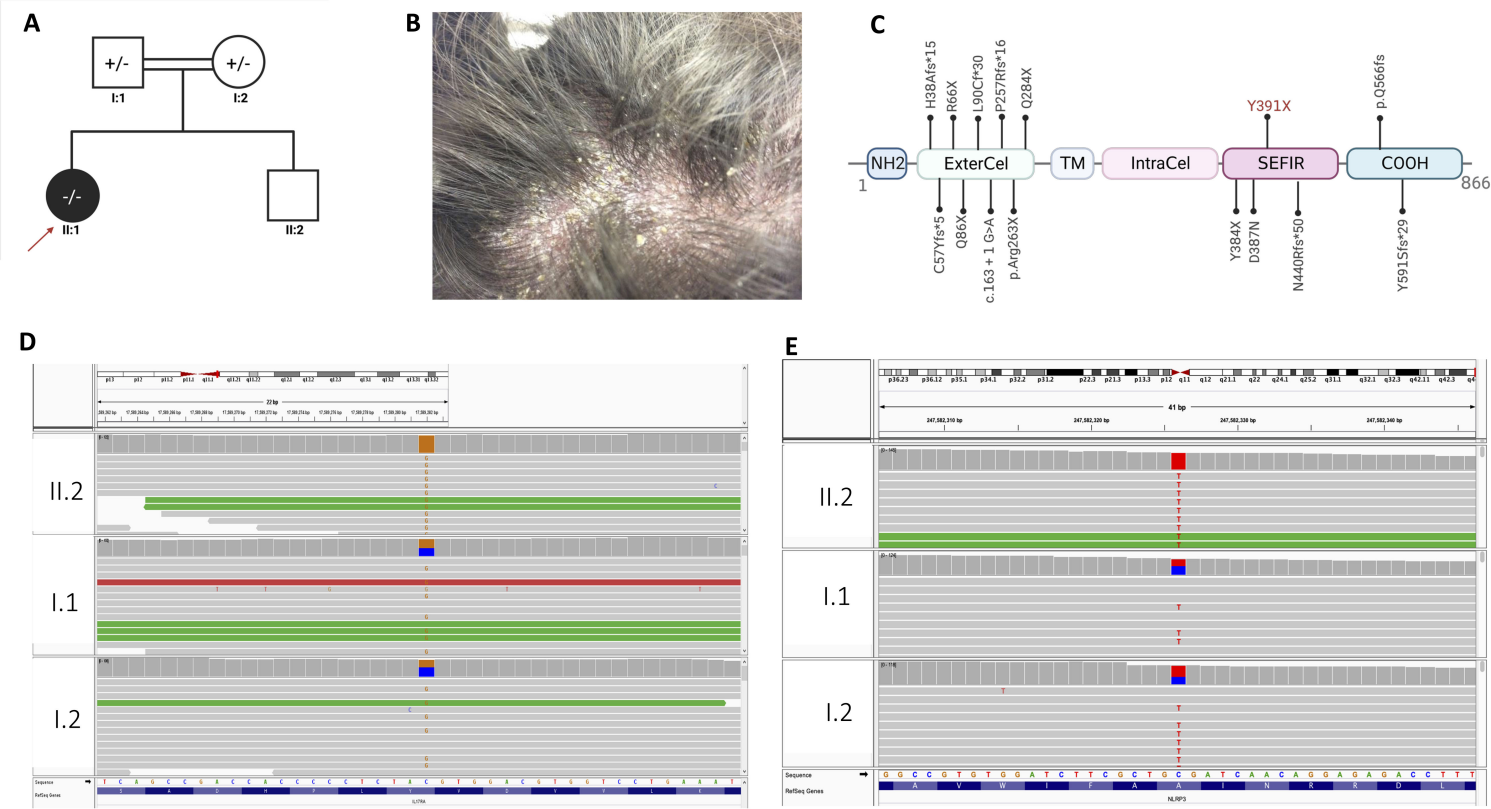
Characterization of the genetic causes of concomitant psoriasis and chronic mucocutaneous candidiasis. **(A)** The pedigree of the family, consanguineous marriage; all members are healthy (highlighted with white color), the proband affected with psoriasis and oral candidiasis is highlighted with red arrowhead. **(B)** Well defined scaly plaques on the scalp of the proband. **(C)** IL17RA protein structure, position of mutation is shown (Y391X) NH2- N terminal domain, ExterCel – Extracellular domain, TM - Transmembrane domain, IntraCel - intracellular domain, SEFIR domain – COOH-C terminal domain. **(D)** IGV visualization of IL17RA; the G > C variant change in the homozygous form for the patient (II.1) and heterozygous form in the mother and father (I.1 and I.2). **(E)** IGV visualization of NLRP3 showing T > C variant change in the homozygous form for the patient (II.1) and heterozygous form in the mother and father (I.1 and I.2). +/- heterozygous -/- homozygous.

The proband II.1 is a 5-year-old female child, the offspring of first-cousin consanguineous Lebanese parents (**Figure 1A**). She presented to the dermatologist at the age of one year with psoriasis on her scalp but no hair loss (**Figure 1B**), along with recurrent erythematous. At the time of presentation, she was not receiving any medical treatment; yet, she had persistent oral mucosal candidiasis, confirmed by a KOH smear showing *candida albicans* growth (**Table 1**). She did not have any other infections, and her immune workup showed unremarkable findings, her White Blood Cell(WBC) count and Automated Differential were within the reference range [Neutrophils[0.8-7.2x10(9)/L], Lymphocytes [1.3-8x10(9)/L], Monocytes [0.1-1.1x10(9)/L], Eosinophils [0-0.7x10(9)/L], Basophils [0-0.2x10(9)/L]. The patient’s parents and brother underwent clinical assessment, which did not reveal any features of psoriasis or oral candidiasis, prompting a genetic workup.

**Table 1:** Genetic and clinical findings of the potential disease-causing variant in the proband.

Genetic Findings												Clinical Findings			
Gene Variants	c.DNA Position	Protein Domain	Coding Impact	CADD Score	Sift	PolyPhen	Predicted Effect (ACMG/MutationTaster)	MAF (gnomAD v2.1.1)	gnomAD Mean Depth Coverage(v2.1)	MAF (ExAC v1.0)	MAF (Lebanese Exomes and other Population Exomes)	Skin	CMC	Auto-immunity	Treatment
*IL17RA* p.Tyr391Ter	c.1173C>G	SEFIR	Nonsense	36	NA	NA	Likely Pathogenic(PVS1**)/** Deletrious (184|16 )	0/141,456 (exomes)	Exomes 69.5 Genomes 32.1	0/60,706	0/72	Psoriasis (scalp, no hiar loss)	KOH smear (*candida albicans*: oral)	–	–
Other Potential disease-causing variants in the Proband															
*HLA-DRB1* p.Gln39Ter	c.115C>T	Beta-1	Nonsense	53	NA	NA	Likely Pathogenic(PVS1**)/** Deletrious (17|183 )	0/72246 (exomes)	Exomes 15.9 Genomes 17.7	1/23358	1)Lebanese28/72 2)KRGDB 708/2852 3)SGDP 16/48				
*NLRP3* p.Ala77Val	c.230C>T	Pyrin	Missense	18.3	Deleterious (0.04)	Possibly damaging (0.533)	Benign/ Benign 23|77	29/249554 (exomes) 2/31326 (genomes)	Exomes 74.9 Genomes 29.2	8/119986	0/72				

CADD, Combined Annotation Dependent Depletion; ACMG, American College of Medical Genetics and Genomics; MAF, Minor Allele Frequency; HGVS, Human Genome Variation Society; KRJBD, Korean Reference Genome Database; SGDP, Simons Genome Diversity Project. NA, not applicable.

### Exome sequencing: a novel bi-allelic deleterious variant in *IL17RA*

The whole exome sequencing results of the patient and her parents yielded an average of 85,000 variants per sample. After applying a stringent filtering approach (see material and methods), 1,882 variants were retained for further analysis. Segregating with the phenotypes and assuming a recessive mode of inheritance, only homozygous variants were included in the first filter, yielding 206 homozygous variants (**Supplementary Figure S1**, see methods). Amongst these, a novel homozygous variant was detected on chromosome 22 (NM_014339.6 c.1173C>G, g.23439C>G) mapping to the *IL17RA* gene, resulting in a premature stop codon (p. Tyr391Ter). This variant was found in a homozygous form in the proband and in a heterozygous manner in her parents (**Figures 1C, D**) (ClinVar#SCV002520326). The variant is classified as pathogenic according to the American College of Medical Genetics and Genomics (ACMG) classification (18), with a Combined Annotation Dependent Depletion (CADD) score of 36 (19). It is predicted to have a deleterious effect with a tree vote score of (184|16), which may cause nonsensemediated decay NMD (-476 AA/more than 10% missing) according to MutationTaster (20). This variant is absent from gnomAD (21), ExAC (22), and our in-house database of 300 Lebanese exomes. While additional variants in *HLA-DRB1* and *NLRP3* genes (**Table 1**, **Figure 1E**) may influence the phenotype(s), we focused on this variant to gain insights into its consequences on IL17RA protein function, as it is implicated in both phenotypes observed in the affected child. Of note, potentially pathogenic variants in genes previously associated with psoriasis such as *PSORS1-13*, MHC, I, *TNF*, and *TRAF3IP2* (23–26), and genes implicated in CMC, including *STAT1*, *AIRE*, *IL17F*, *IL17RC*, and *RORC* (27–29) were absent in our patient.

### Genome-wide transcriptomics identify upregulated pathways related to IL17 pathway

To further elucidate the molecular mechanisms underlying both phenotypes and ascertain the diagnosis, RNA-seq was conducted on lesional skin biopsies from the indexed patient (II.1) and two non-lesional skin samples from healthy controls. The data distribution and sample correlation of the FPKM values are shown in **Supplementary Figures S2A-C**. We detected 1,136 differentially expressed genes (DEGs) with 967 upregulated and 169 genes downregulated when defined by a fold change (FCH) >2 and a false discovery rate (FDR) <0.01 (**Supplementary Table S2**, **Supplementary Figures S2D-F**). **Tables 2A, B** show the top 15 upregulated and downregulated DEGs, respectively. The top upregulated genes include those encoding the S100 calcium-binding proteins (*S100A8, S100A7/A*, and *S100A9*) which exhibited the most significant increase in expression with a log2 fold change of 8.17. Additionally, proteolysis regulation molecules (*SERPINB4, SERPINB3, PI3*), matrix metalloproteinases (*MMP1* and *MMP3*) and chemokines (*CCL18*) were highly upregulated. Conversely, the top downregulated genes include PPAR-fatty acids and lipid metabolism-related genes such as *FADS2*, which exhibited the most substantial decrease in expression, along with *FABP7*, *ACSBG1*, *ELOVL3*, *PM20D*, and *DGAT2L6*, keratinocyte related genes *KRT79 and KRT2*, Serpin Family A Member 12(*SERPINA12*), and (*C1ORF68*).

**Table 2:** The top 15 up/downregulated differentially expressed genes (DEGs).

(a) Upregulated genes										
Symbol	log2 FC	adj. p-value	Chr	Type	Control 1a	Control 1b	Control 2a	Control 2b	Patient a	Patient b
S100A9	8.17693416	0.00021902	1q21.3	protein_coding	3.05837711	3.14590729	2.42068989	2.34885277	10.9267556	10.9140263
ELK2AP	7.96511871	0.00026016	14q32.33	processed_pseudogene	0	0	0.55341359	0.84963104	8.37384393	8.25791579
S100A8	7.49793854	0.00021095	1q21.3	protein_coding	3.22297299	3.20427534	2.6238809	2.56471959	10.4047052	10.3990963
S100A7	6.93517774	6.57E-05	1q21.3	protein_coding	2.83854239	3.31321392	3.16031356	3.20389492	10.1060394	10.0222985
VTRNA1-3	6.8120407	0.00186493	5q31.3	misc_RNA	0	0	0	0	7.9109245	5.7131569
IGLL5	6.52670759	6.94E-05	22q11.22	protein_coding	0.59614896	0.69694372	0.27226929	0.68431516	7.04923868	7.12901506
SERPINB4	6.37927495	5.37E-05	18q21.33	protein_coding	0.22126214	0.51586482	0.16459451	0.14284079	6.67788874	6.60294229
MMP1	5.74151243	5.26E-06	11q22.2	protein_coding	0	0.0746686	0.03089737	0.05283209	5.83466824	5.72755565
CCL18	5.60475791	8.16E-05	17q12	protein_coding	0.24229519	0.10285025	0.44665536	0.50068582	5.97321634	5.88254279
LTF	5.15417654	0.00021095	3p21.31	protein_coding	0.10276682	0.2364816	0.58886788	0.54759987	5.5730412	5.47316997
PI3	5.03872063	1.29E-03	20q13.12	protein_coding	1.40923342	1.35995965	0.35130154	0.47774674	5.8541453	6.02241664
SERPINB3	4.949767	7.71E-06	18q21.33	protein_coding	1.22196367	1.30615139	1.21966728	1.38209172	6.23305783	6.23141321
MMP3	4.63021645	5.26E-06	11q22.2	protein_coding	0	0.0394714	0.06380926	0.08192422	4.71856986	4.63446549
S100A7A	4.57798229	7.71E-06	1q21.3	protein_coding	0.05000666	0.16952099	0.04585433	0.02278808	4.64543075	4.65461884
FABP4	4.36353074	2.00E-02	8q21.13	protein_coding	0.99413001	1.00093745	3.33112332	3.28017687	6.59375861	6.43648669
(b) Downregulated genes										
Symbol	log2 FC	adj. p-value	Chr	Type	Control 1a	Control 1b	Control 2a	Control 2b	Patient a	Patient b
Symbol	log2FC	adj. p-value	Chr	Type	control 1a	control1b	control2a	control2b	Patient a	Patient b
FADS2	-4.7703907	8.16E-05	11q12.2	protein_coding	5.86522105	5.86800763	6.17982352	6.063999	1.23146313	1.21628102
FABP7	-4.3232274	0.00413127	6q22.31	protein_coding	3.93966854	4.10798073	5.39283601	5.26774178	0.25457107	0.45308767
KRT79	-4.1937686	0.0003437	12q13.13	protein_coding	5.40251413	5.10090618	5.70980619	5.52887445	1.24925722	1.23425605
PM20D1	-4.1594922	0.00104106	1q32.1	protein_coding	4.2813941	4.26530186	5.04146641	4.94963298	0.41955291	0.53036045
ALOX15B	-3.5086781	0.00039423	17p13.1	protein_coding	4.28209132	4.27326224	4.62182917	4.69598825	0.92222602	0.9970033
GAL	-3.3676949	0.00101649	11q13.2	protein_coding	3.57618349	3.41043202	4.00452836	4.08137372	0.48013481	0.3207342
THRSP	-3.2907684	8.16E-05	11q14.1	protein_coding	4.79466019	4.64582845	4.87170568	4.76263584	1.51639343	1.43948484
C1orf68	-3.1050519	0.00052195	1q21.3	protein_coding	4.17594769	4.30013837	3.91141356	3.95247336	1.13255052	0.82733208
DGAT2L6	-3.0635369	0.01422656	Xq13.1	protein_coding	2.41673978	2.3046483	3.78686815	3.81767442	0	0.03589145
ACSBG1	-3.0386056	0.00021902	15q25.1	protein_coding	3.89762767	3.72908503	4.02575528	3.92523991	0.76281993	0.94882284
AADACL3	-3.021471	1.67E-04	1p36.21	protein_coding	2.88022708	2.95466534	3.13082711	3.12016446	0	0
SERPINA12	-2.9762025	2.92E-03	14q32.13	protein_coding	4.68048752	4.66440319	3.89239103	3.85887618	1.29767859	1.29799546
ELOVL3	-2.8620087	8.67E-03	10q24.32	protein_coding	3.06764216	2.91597018	4.13341557	4.05660954	0.50638766	0.85641368
AWAT2	-2.856141	2.64E-04	Xq13.1	protein_coding	2.71101026	2.8964288	3.03151695	3.01427316	0.08591463	0.02841794
KRT2	-2.8135481	3.42E-03	12q13.13	protein_coding	7.72292363	7.73023915	8.49033346	8.5499502	5.27465304	5.34497404
IL37	-2.6347421	2.01E-03	2q14.1	protein_coding	2.9632614	3.2455489	2.47201459	2.60986604	0.25289398	0.12296739

RNAseq performed in technical duplicates.

**Figure 2:**
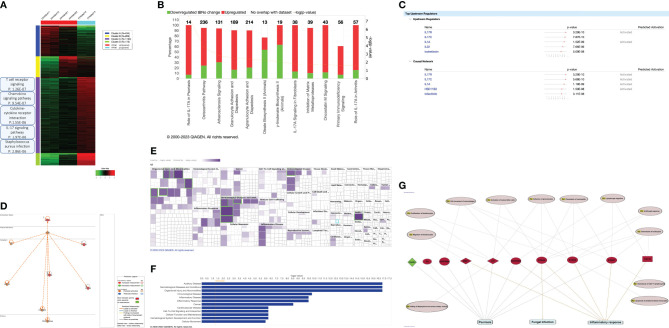
Transcriptional profiling of the affected psoriatic skin biopsy. **(A)** Heatmap of different enriched clusters in distinct biological pathways (based on kmeans and GO biological process (patient vs control). **(B)** The Top 12 Canonical pathways ranked from the most significant according to the – log(p-value). For the complete list, check **Supplementary Table S4**. **(C)** The top 5 upstream regulators and casual network in the dataset (patient vs control). For the complete list, check **Supplementary Table S5**. **(D)** The master upstream regulator: IL17R. **(E)** Heatmap of the top disease and function categories. The top 5 categories (based on the p-value) are highlighted with green boxes. **(F)** The top 12 disease and function categories are shown as a bar chart. **(G)** Shared molecules and disease function annotation between psoriasis and CMC. Psoriasis, fungal infection, and inflammatory shares 9 molecules depicted in red, LTF, MMP1, S100A7, S100A8, S100A9, SERPINB3, SERPINB4. Each molecule is associated with disease function annotation in pink circles.

We identified 39 enriched pathways with an adjusted p-value (adj.p-value) of <0.05 (**Figure 2A**; **Supplementary Table S3**). Among the top 15 enriched pathways (**Table 3**), T cell receptor signaling (adj.p-value 1.26E-07), chemokine signaling (adj.p-value 9.54E-07), cytokine-cytokine receptor (adj.p-value 1.55E-06), Staphylococcus aureus infection (adj.p-value 2.86E-06), and notably the IL17 signaling pathway (adj.p-value 1.97E-06) were upregulated (**Figure 2B**).

**Table 3:** The top 15 enrichment clustered genes based on k- means (patient vs control).

Cluster	adj. p-value	nGenes	Pathways	Genes
C	4.82E-40	58	Systemic lupus erythematosus	HIST2H3C CTSG FCGR2A FCGR3B HIST1H2AE HIST1H2AD H2AFZ HIST1H2BB HIST2H3A HIST2H2BF HIST2H4B HIST2H3D C1R C1S HIST2H2AA4 HIST1H4I HIST1H2AI HIST1H2AJ HIST1H2AL HIST1H2AB HIST1H2AM HIST2H2AA3 HIST1H2BG HIST1H2BL HIST1H2BN HIST1H2BM HIST1H2BF HIST1H2BE HIST1H2BH HIST1H2BI HIST1H2BC HIST1H2BO HIST1H3A HIST1H3D HIST1H3C HIST1H3E HIST1H3I HIST1H3G HIST1H3J HIST1H3H HIST1H3B HIST1H4A HIST1H4D HIST1H4F HIST1H4K HIST1H4J HIST1H4C HIST1H4H HIST1H4B HIST1H4L HIST1H2AH ACTN1 HIST1H3F HIST1H2AG HIST1H2BJ CD28 CD80 CD86
C	1.88E-30	57	Alcoholsim	HIST2H3C FOSB GNG5 GNG10 HIST1H2AE HIST1H2AD H2AFZ HIST1H2BB HDAC1 HIST2H3A MAOB HIST2H2BF HIST2H4B HIST2H3D HIST2H2AA4 CALM1 CALM2 HIST1H4I HIST1H2AI HIST1H2AJ HIST1H2AL HIST1H2AB HIST1H2AM HIST2H2AA3 HIST1H2BG HIST1H2BL HIST1H2BN HIST1H2BM HIST1H2BF HIST1H2BE HIST1H2BH HIST1H2BI HIST1H2BC HIST1H2BO HIST1H3A HIST1H3D HIST1H3C HIST1H3E HIST1H3I HIST1H3G HIST1H3J HIST1H3H HIST1H3B HIST1H4A HIST1H4D HIST1H4F HIST1H4K HIST1H4J HIST1H4C HIST1H4H HIST1H4B HIST1H4L HIST1H2AH HIST1H3F HIST1H2AG HIST1H2BJ CREB5
C	1.42E-16	44	Viral carcinogenesis	CDK4 CDKN1A YWHAQ CCR5 EGR3 GTF2A2 HIST1H2BB HDAC1 JAK3 JUN LYN HIST2H2BF PMAIP1 HIST2H4B VDAC3 HIST1H4I HIST1H2BG HIST1H2BL HIST1H2BN HIST1H2BM HIST1H2BF HIST1H2BE HIST1H2BH HIST1H2BI HIST1H2BC HIST1H2BO HIST1H4A HIST1H4D HIST1H4F HIST1H4K HIST1H4J HIST1H4C HIST1H4H HIST1H4B HIST1H4L PIK3R3 ACTN1 CCNA2 CCND2 HIST1H2BJ ATP6V0D1 CREB5 CDK1 CDC20
B	4.44E-09	16	Influenza A	IRF9 HLA-DRA HLA-DRB1 HLA-DRB5 CXCL10 CIITA MX1 OAS1 OAS2 OAS3 EIF2AK2 IFIH1 STAT1 STAT2 TNFSF10 RSAD2
C	4.91E-09	28	Cell adhesion molecules (CAMs)	CDH3 CD226 CLDN4 VCAN CTLA4 TIGIT CD274 ICOS ICAM1 ITGA4 ITGAL ITGB2 PDCD1 SDC2 SELE SELL SELP SELPLG VCAM1 PDCD1LG2 MPZL1 CD4 CD6 CD8A CD8B CD28 CD80 CD86
C	1.93E-08	21	Rheumatoid arthritis	TCIRG1 TNFSF13B CTLA4 CTSK ICAM1 IL1B ITGAL ITGB2 JUN ATP6V0B ACP5 CCL2 CCL5 CCL20 TLR2 ATP6V0E1 ATP6V0D1 ATP6V1F CD28 CD80 CD86
C	3.70E-08	27	Phagosome	TCIRG1 TUBA1B SEC61B CORO1A CTSS FCGR2A FCGR2B FCGR3B SEC61G CD209 ITGB2 M6PR MRC1 ATP6V0B ACTB NCF1 THBS1 TLR2 C1R CALR TUBB6 TUBA1C MARCO ATP6V0E1 ATP6V0D1 ATP6V1F CD36
C	4.07E-08	28	Necroptosis	ALOX15 FTH1 FTL GLUL PYCARD HIST1H2AE HIST1H2AD H2AFZ BIRC2 HSP90AA1 IFNGR1 IFNGR2 IL1B JAK3 PYGL BID TNFAIP3 HIST2H2AA4 VDAC3 HIST1H2AI HIST1H2AJ HIST1H2AL HIST1H2AB HIST1H2AM HIST2H2AA3 CASP1 HIST1H2AH HIST1H2AG
C	5.83E-08	15	Malaria	HBA1 HBA2 ICAM1 IL1B ITGAL ITGB2 KLRB1 CCL2 SDC2 SELE SELP THBS1 TLR2 VCAM1 CD36
C	1.26E-07	21	T cell receptor signaling pathway	RASGRP1 CDK4 CTLA4 ICOS ITK JUN LCK NFKBIE PDCD1 VAV1 ZAP70 CARD11 PIK3R3 BCL10 CD3G CD247 CD4 CD8A CD8B CD28 GRAP2
C	9.54E-07	28	Chemokine signaling pathway	CXCR6 CCL27 CCR1 CCR5 DOCK2 FGR PIK3R5 GNG5 GNG10 CXCL2 HCK ITK JAK3 LYN ARRB2 PLCB2 PREX1 CXCL16 RAP1B CCL2 CCL5 CCL17 CCL20 NCF1 CCR2 VAV1 WAS PIK3R3
C	1.55E-06	36	Cytokine-cytokine receptor interaction	CXCR6 TNFSF13B CCL27 IL17F CCR1 CCR5 CSF2RA CSF2RB IL36RN TNFRSF21 CXCL2 IFNGR1 IFNGR2 IL1B IL1RN IL2RA IL4R IL10RA IL12RB2 IL13RA1 IL21R TNFRSF12A CXCL16 CCL2 CCL5 CCL17 CCL20 CRLF2 TNFRSF1B CCR2 TNFRSF4 TNFRSF6B IL1RL1 CD4 CD27 IL27RA
D	1.97E-06	9	IL-17 signaling pathway	FOS CXCL1 MMP1 MMP3 MMP9 S100A7 S100A8 S100A9 FOSL1
C	2.86E-06	15	Staphylococcus aureus infection	FCGR2A FCGR2B FCGR3B FPR1 ICAM1 ITGAL ITGB2 DEFB103B MASP1 PTAFR SELP SELPLG C1R C1S C3AR1
B	3.53E-06	13	NOD-like receptor signaling pathway	IRF9 GBP4 GBP5 GBP1 GBP2 GBP3 ITPR1 OAS1 OAS2 OAS3 STAT1 STAT2 PSTPIP1

Interestingly, we found that the most significant canonical pathway is IL17A in Psoriasis [pvalue: 2.65E-08, -log (p-value: 7.58)] amongst 16 canonical pathways identified by IPA with a p-value of ≤ 0.05 (**Figure 2B**; **Supplementary Table S4**).

IL17R emerged as the master upstream regulator (p-value 3.29E-10) with a predicted activation in the pathway indicated by a z-score of 2, among 566 upstream regulators with a of p-value ≤ 0.05. (**Figures 2C, D**; **Supplementary Table S5**).

In terms of diseases categories, dermatological diseases (with a p-value between 1.44E-17 and 4.21E-02) were among the second top categories out of 73 identified (**Figures 2D, E**; **Supplementary Table S6**). Among these, 877 disease function annotations were identified with a p-value ≤ 0.05 (**Supplementary Table S7**). Strikingly, under the “dermatological diseases” category, psoriasis emerged as the top annotated disease function with a p-value of 2.94E-12, involving 9 corresponding molecules (**Supplementary Table S7**). We conducted additional analysis to identify and annotate molecules and biological functions that are common to psoriasis, fungal infection (associated with CMC), and inflammatory response. This analysis is detailed in **Figure 2G** and **Supplementary Table S8**.

### Detection of normal levels of IL17RA proteins in the patient

Moreover, to gain better insight into this increased IL17 pathway activity, we quantified the presence of the protein in the patient’s PBMCs.

**Figure 3:**
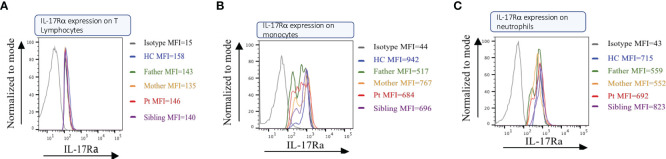
IL17RA expression in the patient/parents/sibling’s peripheral blood mononuclear cells (PBMC). Cell sorting in Lymphocytes **(A)**, Monocytes **(B)**, and Neutrophils **(C)** show similar expressions between the patient and her non-affected family members.

We used flow cytometry on PBMCs from the patient, her sibling, and her parents, using a specific monoclonal antibody against the IL17RA protein. Results showed that the patient (II.1) expresses normal levels of IL17RA on her T lymphocytes, monocytes, and neutrophils (**Figure 3**). This suggests that the mutation produces a stable truncated protein, which may be capable of homodimerizing and potentially enhancing the IL17RA downstream pathway. These findings prompted us to assess the structure and function of the mutated protein *in vitro*.

### The IL17RA mutant protein is localized at the cell membrane and maintains the interation with IL17RC and IL17RD

To assess the impact of the Y391* variant on the structural and functional properties of IL17RA, site-directed mutagenesis was performed on human *IL17RA* cDNA cloned into an HA-tagged expression vector. The generated plasmid was transfected into HEK293 and HaCat cells to examine the localization of the mutant protein and compared to the wild-type IL17RA (Wt).

**Figure 4:**
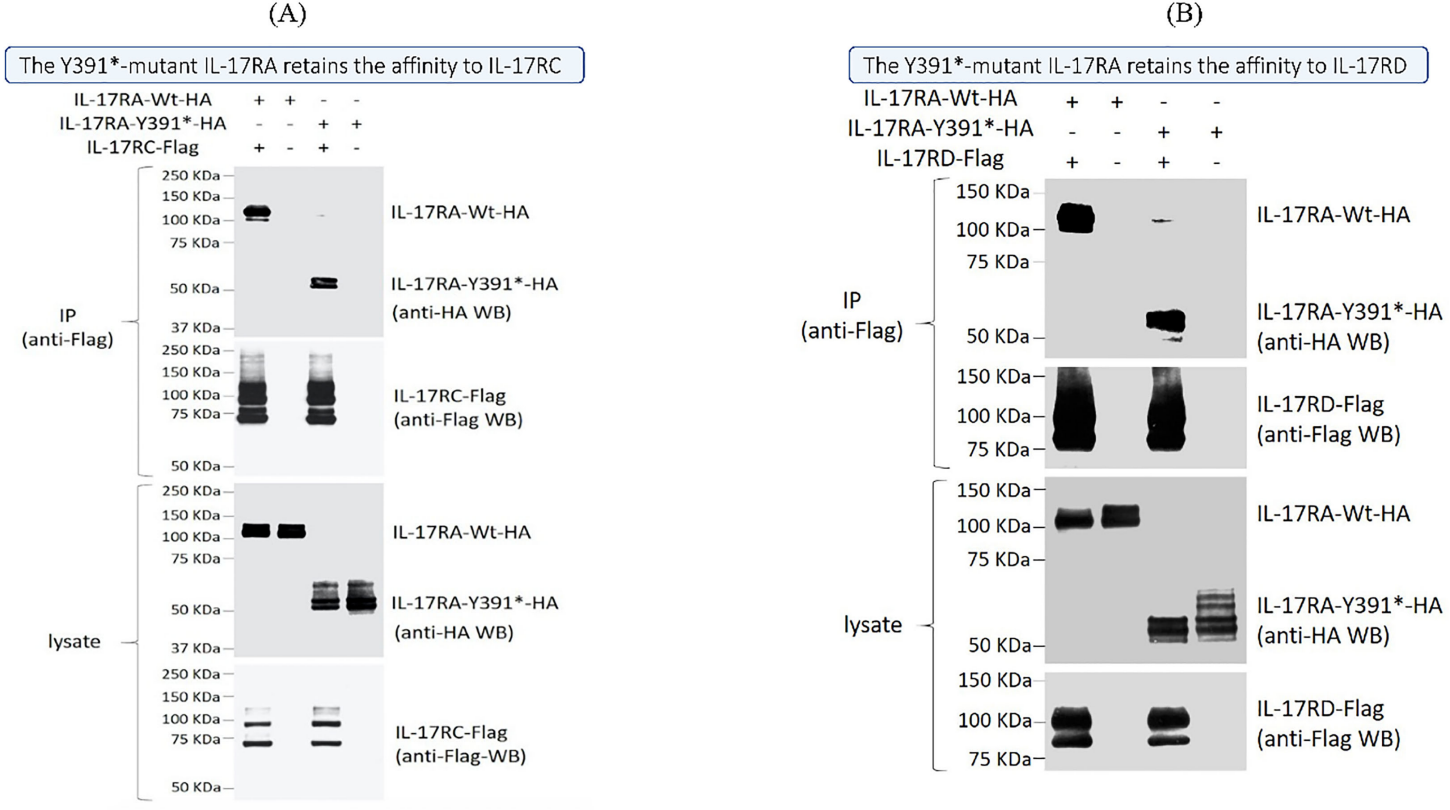
Physical interaction between the IL17RA wild type and mutated protein and their partners IL17RC and IL17RD. Co-immunoprecipitation assays using transiently expressed Flag-tagged IL17RC **(A)** or IL17RD **(B)** along either an HA-tagged wild-type (Wt) or mutant IL17RA (IL17RA Y391*) showed similar levels of interaction. Immunoprecipitations (IP) were done using an anti-Flag antibody, and both anti-HA and anti-Flag antibodies are sued to reveal the bands on western blots.

Immunofluorescence studies revealed that both Wt-IL17RA protein and mutant protein IL17RA Y391* were localized to the cell membrane in both HEK293 and HaCat cells (**Supplementary Figures S2A, B**). To assess the physical interaction with IL17RC/D, Co-immunoprecipitation (IP) assays were performed. The results showed that the mutant p.Y391* retained its capability to bind to both IL17RC and IL17RD proteins. This finding confirms a potential deregulated signaling pathway that could contribute to the underlying psoriatic phenotype (**Figures 4A, B**).

## Discussion

Psoriasis and CMC share an intriguing undercurrent immune dysfunction, chronic inflammation, and susceptibility to perturbations in IL17-mediated signaling. The IL17RA plays a pivotal role in Th17 cell differentiation and downstream signaling, significantly influencing the inflammatory pathways in both conditions. In this study, we have detected a novel deleterious mutation in *IL17RA* causing a dual phenotype, resulting in a functional receptor and demonstrating the complexity of receptor-ligand interaction in the IL17 family.

Mutations in the *IL17RA* gene have been previously linked to CMC and, in some cases, other skin abnormalities (**Table 4**) (10). However, our indexed patient developed psoriasis and CMC from the age of one, effectively ruling out medication-induced side effects as a cause, given the absence of systemic medication use.

**Table 4:** Reported mutations in *IL17RA* associated with CMC or skin anomalies.

Mutation	Pediatric Patient	Country	Consanguinity	Phenotype	Institution
H38Afs*15	Yes	Saudi Arabia	Yes	Oral Mucosal Candidiasis	IMAGINE
C57Yfs*5	Yes	Turkey	Yes	Oral Mucosal Candidiasis +Skin Folliculitis	IMAGINE
R66X	Yes	Japan	Yes	Oral Mucosal Candidiasis +Skin Folliculitis	IMAGINE
Q86X	Yes	Turkey	Yes	Oral Mucosal Candidiasis +Skin Folliculitis	IMAGINE
L90Cf*30	Yes	Saudi Arabia	Yes	Oral Mucosal Candidiasis +Skin Folliculitis	IMAGINE
c.163 + 1 G>A	Yes	Algeria	Yes	Oral Mucosal Candidiasis	IMAGINE
P257Rfs*16	Yes	Turkey	Yes	Oral Mucosal Candidiasis +Skin Folliculitis	IMAGINE
p.Arg263X	Yes	Turkey	Yes	Oral Mucosal and genital Candidiasis+Eczema	Hacettepe University School of Medicine
Q284X	Yes	Morocco	Yes	Oral Mucosal Candidiasis +Skin Folliculitis	IMAGINE
Y384X	Yes	Argentina	Yes	Oral Mucosal Candidiasis +Skin Folliculitis	IMAGINE
D387N	Yes	Turkey	Yes	Oral Mucosal Candidiasis +Skin Folliculitis	IMAGINE
Y391X (This study)	Yes	Lebanon	Yes	Oral Mucosal Candidiasis+Psoriasis	AUB
N440Rfs*50	Yes	Turkey	Yes	Oral Mucosal Candidiasis +Skin Folliculitis	IMAGINE
p.Q566fs	Yes	N/A	No	Mucocutaneous Candidiasis+ Eczema	The Hospital for Sick Children
Y591Sfs*29	Yes	Turkey	Yes	Oral Mucosal Candidiasis +Skin Folliculitis	IMAGINE

While this is the first report linking a mutation in *IL17RA* to psoriasis in humans, a psoriasis-like skin alteration phenotype and immunodeficiency has been reported in cattle caused by recessively inherited frameshift variant in *IL17RA* (XP_015316220.2: p. Cys61AlafsTer62), resulting in a loss-of-function LOF effect (30). The reported loss of function *IL17RA* mutation in cattle might suggest a phenotype like psoriasis. One could argue, however, that alternative splicing might generate an N-terminal truncated form with potentially greater potency, a hypothesis we propose for our truncated protein.

**Figure 5:**
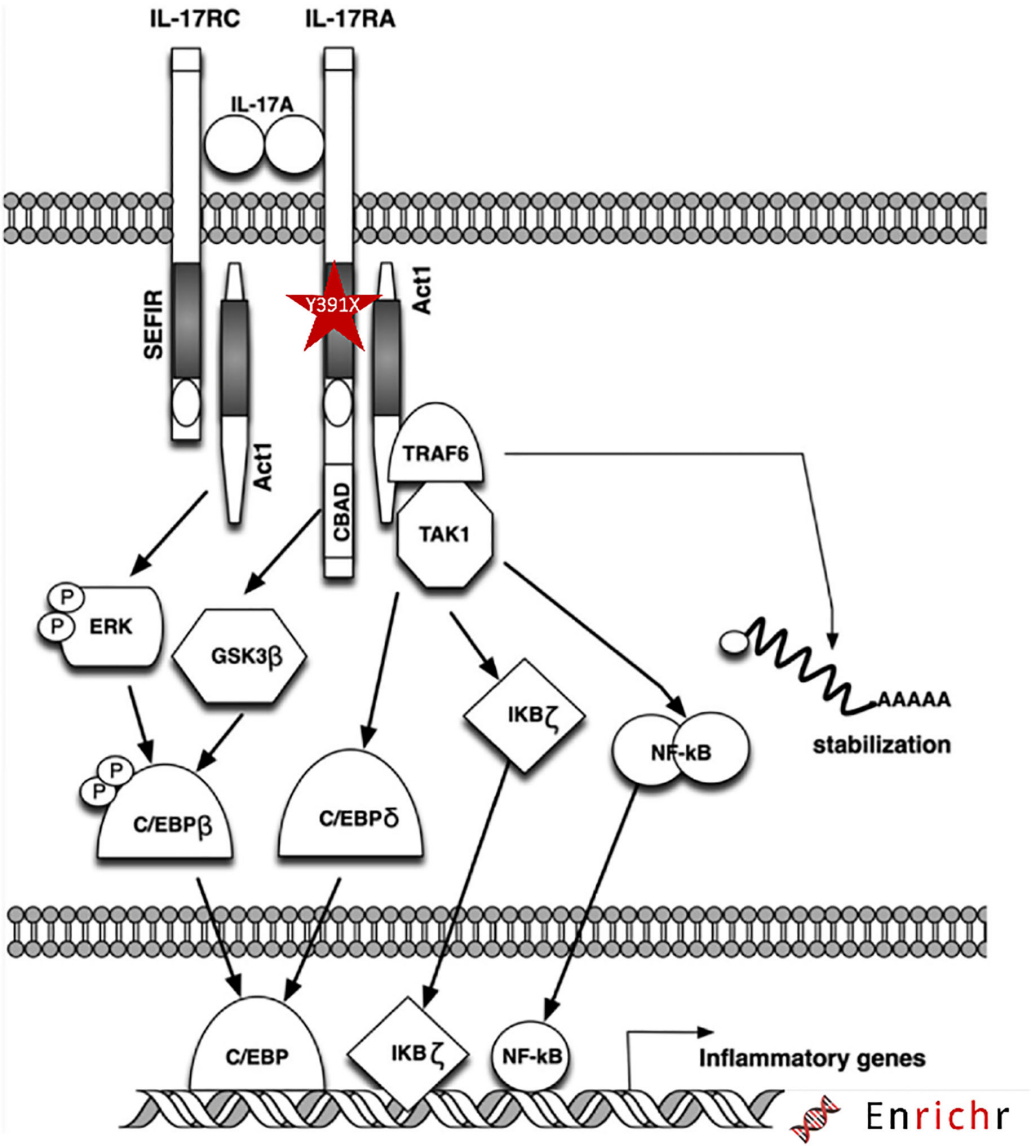
Schematic diagram of the IL17RA mutation and downstream signaling pathway. The mutation is highlighted with red star within the SEFIR domain of IL17RA.

Our mutation results in a presumably truncated receptor lacking the SEFIR domain, an essential component for its downstream activity (**Figure 1C**). This domain is required for docking to the nuclear factor activator protein 1 (ACT1), encoded by *TRAF3IP2*, thereby activating proinflammatory pathways (**Figure 5**) involving the different players mainly MAPK, NF-κB, and C/EBP (31, 32).

The complexity of ascertaining a mode of action is intimately related to the broad spatiotemporal expression of IL17RA and its network of intracellular and extracellular partners. We have previously shown that a missense mutation in *TRAF3IP2* resulted in a partial defect of physical interaction with IL17RA but would preserve or potentially enhance its interaction with TRAF6. This pleiotropic and wide effect spans from scarring alopecia to folliculitis decalvans in affected patients (16). Others have highlighted a seemingly contradictory effect between innate and adaptive immunity in a *TRAF1* polymorphism, which is associated with reduced expression of the protein but paradoxically leads to a gain of function that leads to rheumatoid arthritis (33). This model proposes that distinct TRAF1 partners are present in T-cells and monocytes, leading to a differential contextual effect that shapes the phenotype. It has been proposed that TRAF1’s role in restraining innate immune inflammatory signaling surpasses its function in maintaining TNFR superfamily signaling in T-cells (34). It was also demonstrated that IL17RA mobilizes, attracts, and triggers neutrophils (35). This connection between innate and adaptive immunity might provide additional insight into the intricacies of the observed phenotype.

Our findings show preserved expression of the IL17RA variant in the patient’s PBMCs and *in vitro* analysis, along with normal membrane localization and functional heterodimerization with IL17RC and IL17RD (**Figures 3**, **4**). Paradoxically, despite harboring this truncated form of the protein, transcriptomic analysis showed a strong activation of the IL17A/RA axis, bearing the signature of a psoriatic phenotype (**Figure 2B**). Notably, we discovered that IL17R serves as a unifying genetic factor, linking these seemingly disparate conditions by modulating immune responses, inflammation, and tissue integrity (**Figures 2C, D**). A loss of function of *IL17RA* in keratinocytes could trigger a feedback mechanism resulting in hyperproduction of IL17 in the skin and recruitment or expansion of more Th17 cells, as observed in mice. Indeed, it was previously shown that *Il17ra* knockout mice exhibit significantly elevated levels of Th17- and IL17A-producing dermal γδ T cells in the skin, indicating the role of IL17RA in regulating the size of these populations in the skin (36).

Moreover, recent insights into the structure and function of the IL17 signaling pathway have highlighted the formation of a hexametric complex and a two-faced cytokine signature with unique receptor recognition properties. This includes IL17A or F binding to receptors IL17RA/C or ILRA/D, respectively. These structural considerations may provide a plausible explanation for the dual phenotype observed in our indexed patient (37). The hyperactivity of the IL17A pathway in our study results in overstimulation of inflammatory responses, typical of conditions like psoriasis, where excessive IL17 signaling leads to chronic skin inflammation. In CMC, the hyperactivity could be paradoxical: while IL17 normally confers protection against *Candida*, excessive or dysregulated signaling might disrupt the delicate balance of immune regulation, potentially contributing to immune exhaustion, impaired recruitment of effective immune cells, or altered antimicrobial responses, allowing Candida to persist.

Thus, treatments targeting IL-17 pathway have gained prominence in managing psoriasis. Brodalumab’s stands out among IL-17 inhibitors due to its unique approach of targeting IL-17RA directly, unlike other IL-17 inhibitors like secukinumab and ixekizumab that target individual cytokines. By binding to the receptor itself, Brodalumab prevents the activity of several IL-17 cytokines, including IL-17A, IL-17E, and IL-17F, thereby disrupting downstream inflammatory signals at its source (13, 14, 38).

However, the broader inhibition achieved by Brodalumab carries a greater risk of side effects like Candida infections. This is because IL-17 plays a critical role in immune defense against fungal pathogens. Remarkably, inhibiting IL-17 signaling with brodalumab might lead to changes in the production or activity of IL-22. Since IL-22 is involved in maintaining epithelial barrier integrity and defending against extracellular pathogens often in collaboration with IL-17, any modulation in the Th17 pathway could influence these processes indirectly. Brodalumab’s effect on IL-22 activity could theoretically weaken mucosal immunity, resulting in fungal infections like *Candida albicans* (39). Indeed, mice lacking both IL-17 and IL-22 receptors exhibit significantly higher fungal loads and more severe symptoms compared to those lacking only one of the receptors, indicating that these cytokines work together to enhance antifungal defenses (40). This warrants the importance of further investigation of the relationship between IL-17 and IL-22.

As a result, treatments are now shifting focus toward IL-23 inhibitors, as studies have shown that the incidence of Candida infections is higher in patients treated with IL-17 inhibitors compared to those receiving IL-23 inhibitors. IL-23 inhibitors act upstream in the inflammatory cascade and do not directly suppress IL-17 activity. This indirect modulation allows for the preservation of mucosal immunity, resulting in a lower likelihood of Candida infections (3, 41).

Finally, the complexity in deciphering a genotype/phenotype correlation is further exacerbated by the findings of additional SNPs in other genes involved in the immune response. The NLRP3 inflammasome, a hallmark of the innate immune sensing pathway, and as such could be relevant to the CMC phenotype observed in our indexed patient. Previous reports have linked mutations in *NLRP3* to familial cold autoinflammatory syndrome 1 (CAIS1) and muckle-wells syndrome (MWS), both inherited in an autosomal dominant manner (42). In our case, the bi-allelic variant NP_001073289.1: p. Ala77Val in *NLRP3* exhibits an exceptionally low minor allele frequency (MAF). Loss-of-function mutations in *NLRP3* are associated with increased susceptibility to infections, potentially linking this variant to the CMC phenotype. In contrast, gain-of-function mutations have been associated with susceptibility to auto-immune diseases like arthritis and psoriasis (43). Nevertheless, a detailed mechanistic understanding of *NLRP3* mutations in human disease remains unclear, highlighting the need for an in-depth investigation of protein interactions. We also identified a variant p. Gln39Ter in *HLA-DRB1*, but we excluded as it was detected in normal individuals within our in-house exome database and in two other large-scale genome population studies (Korean and African projects, last accessed in May 2024 (https://www.ncbi.nlm.nih.gov/snp/rs9269957#seq_hash) (44, 45).

In summary, our study uncovers a novel deleterious mutation in *IL17RA* that maintains a functional receptor, highlighting the intricate interactions within the IL17 family. By identifying psoriasis as a novel phenotype alongside CMC for being associated with *IL17RA* variants, our findings expand the current understanding of IL17RA’s diverse roles in health and diseases. The strength of this study lies in being the first case to document this phenomenon with an in-depth analysis. Nonetheless, the results require validation through larger patient cohort for broader applicability. Comprehensive insights into IL17RA signaling pathways could pave the way for developing more effective IL17 receptor inhibitors with higher efficacy and reduced side effects, facilitating individualized therapy and overcoming treatment resistance.

## Data Availability

The original contributions presented in the study are included in the article/**Supplementary Materials**, further inquiries can be directed to the corresponding authors.

## Supplementary material

Supplementary Figure 1 Dataflow chart of the variant curation.

Supplementary Figure 2 Results of RNASeq analysis (Data distribution and sample correlation of transformed data and expression in technical duplicate). **(A)** Scatter plot of transformed expression in patient and controls line represent R:0.995 **(B)**Distribution of transformed data in controls and patients shown in boxplot **(C)** Density of transformed data in respect to expression values in the patient and controls **(D)** Number of differentially expressed genes (1136 genes:967 d 169 downregulated) **(E)** Volcano plot of differentially expressed genes **(F)** Heatmap of hierarchical clustering of the most 1000 variable genes.

Supplementary Figure 3 Results of expression studies (Wild-type-Wt) or p.Y391* mutant (Mut) of IL17RA protein in HEK293 and HaCat cultured cells. (A)Wild-type (Wt) and p.Y391* mutant (Mut) IL17RA protein with an N-terminal HA-tag was overexpressed in HEK293T cells, the expression patterns were analyzed by indirect immunofluorescence (IIF) with anti-HA and anti-pan-cadherin antibodies. The results showed that both the Wt-IL17RA protein and Mut IL17RA p.Y391* protein were localized around the cell membrane. (B) Wild-type (Wt) and p.Y391* mutant (Mut) IL17RA protein with an N-terminal HA-tag was overexpressed in HaCat cells. The expression patterns were analyzed by indirect immunofluorescence (IIF) with anti-HA and Hoechst staining. Both the wild type-IL17RA and mutant IL17RA p.Y391* proteins were localized around the cell membrane.
