# Supplementary material for: Deciphering the role of IL17RA in psoriasis and chronic mucocutaneous candidiasis: shared pathways and distinct manifestations
Source: Front Immunol. 2025 Jan 20;15:1516408. doi: 10.3389/fimmu.2024.1516408 (PMC11796622; doi:10.3389/fimmu.2024.1516408)

Supplementary Figure 1

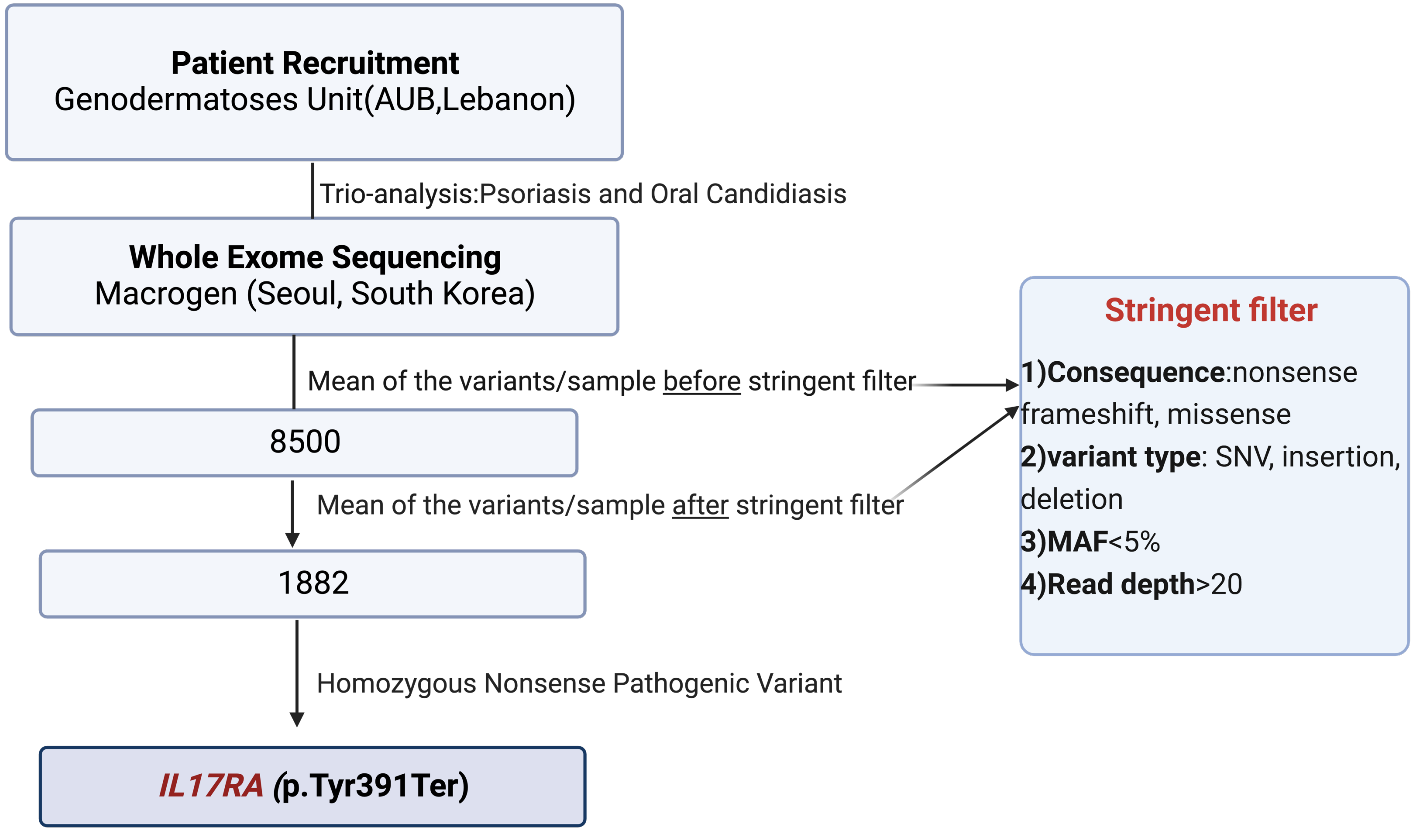

Supplementary Figure 2 (A)

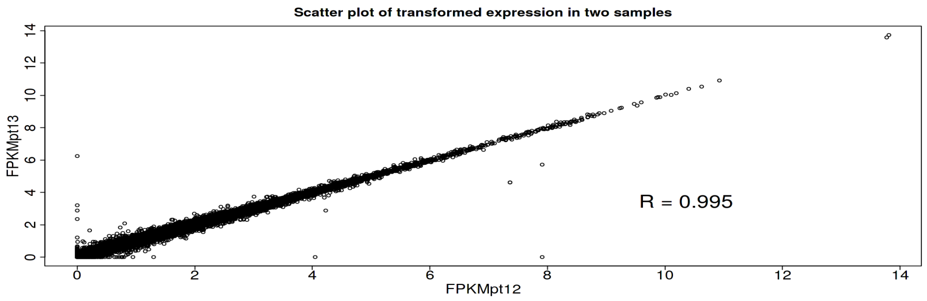

Supplementary Figure 2 (B)

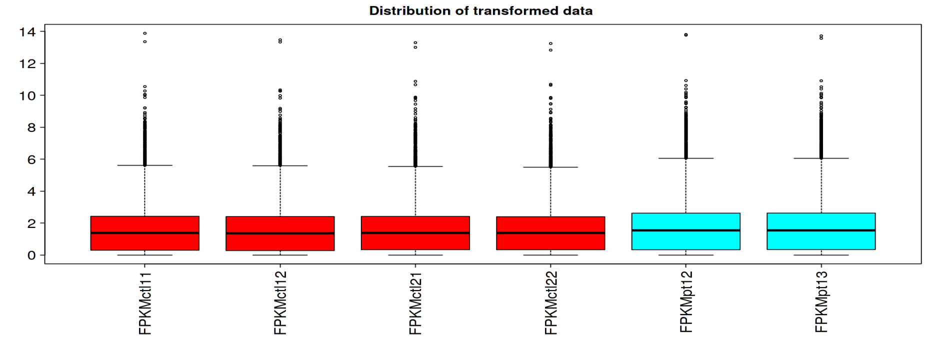

Supplementary Figure 2 (C)

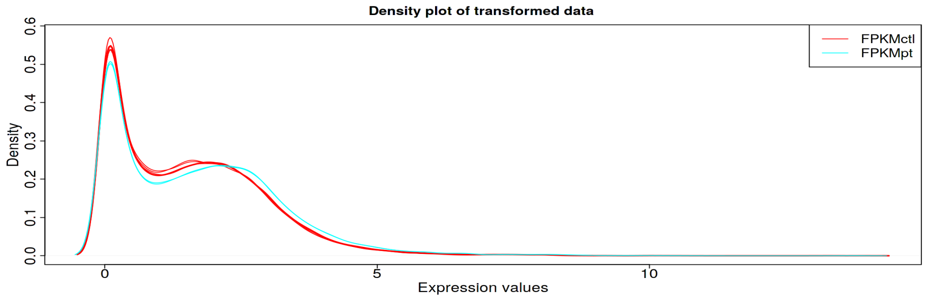

Supplementary Figure 2 (D)

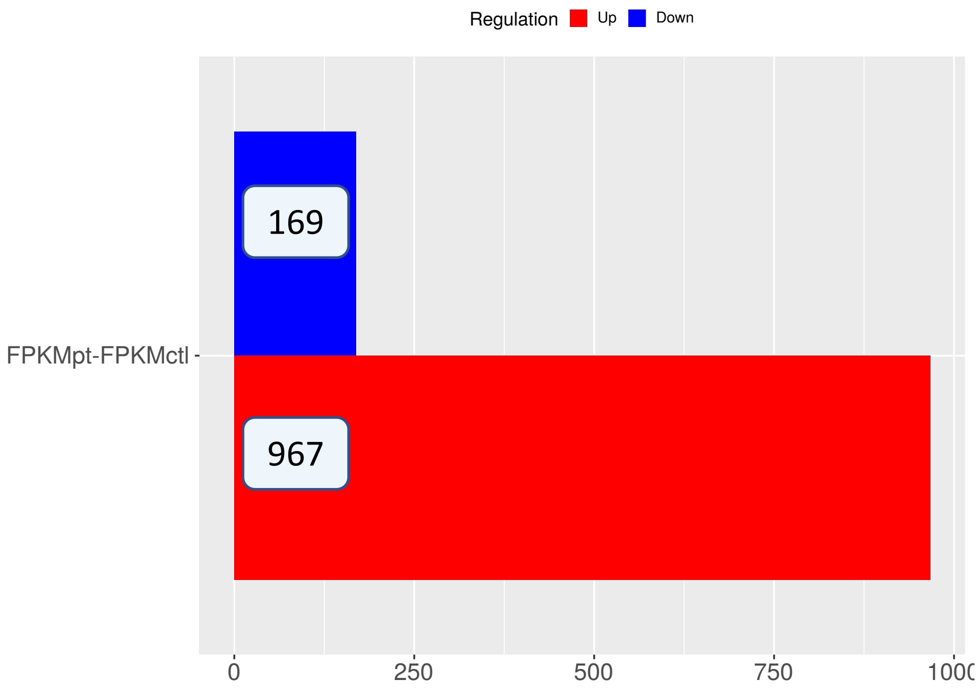

Supplementary Figure 2 (E)

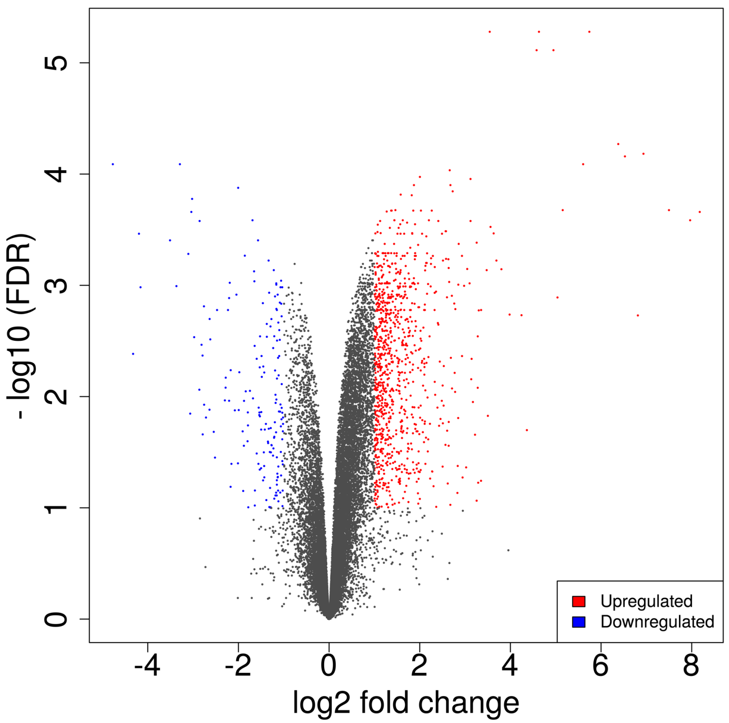

Supplementary Figure 2 (F)

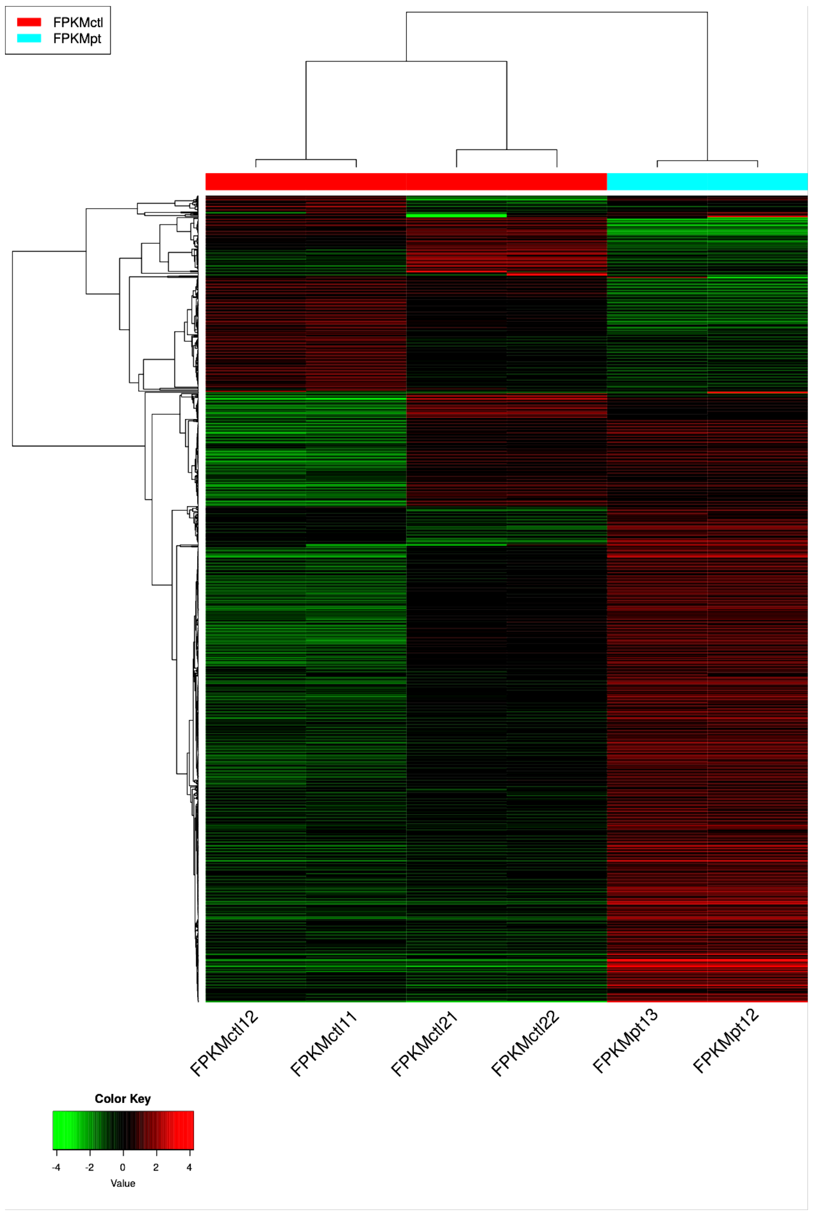

Supplementary Figure 3 (A)

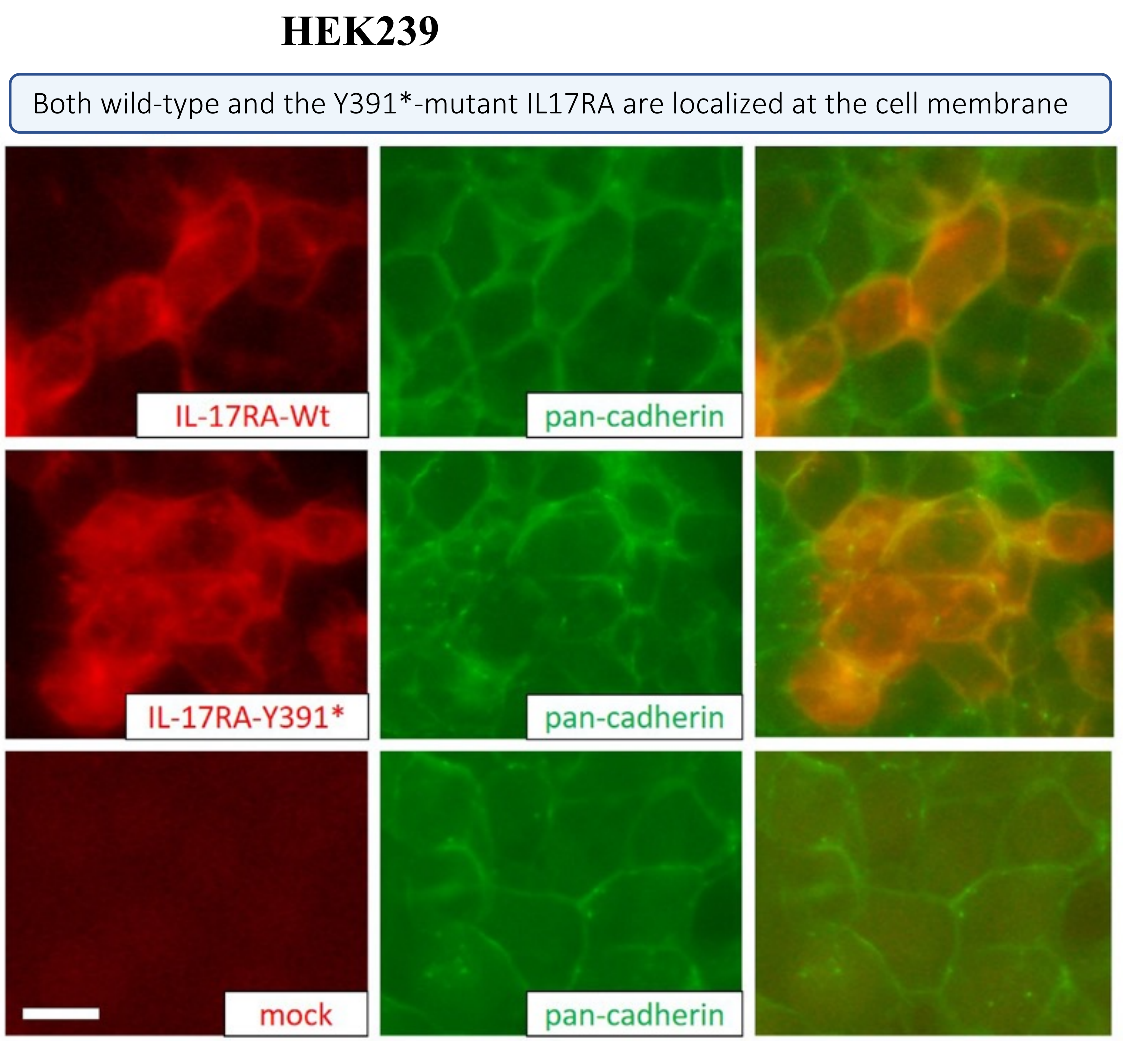

Supplementary Figure 3 (B)

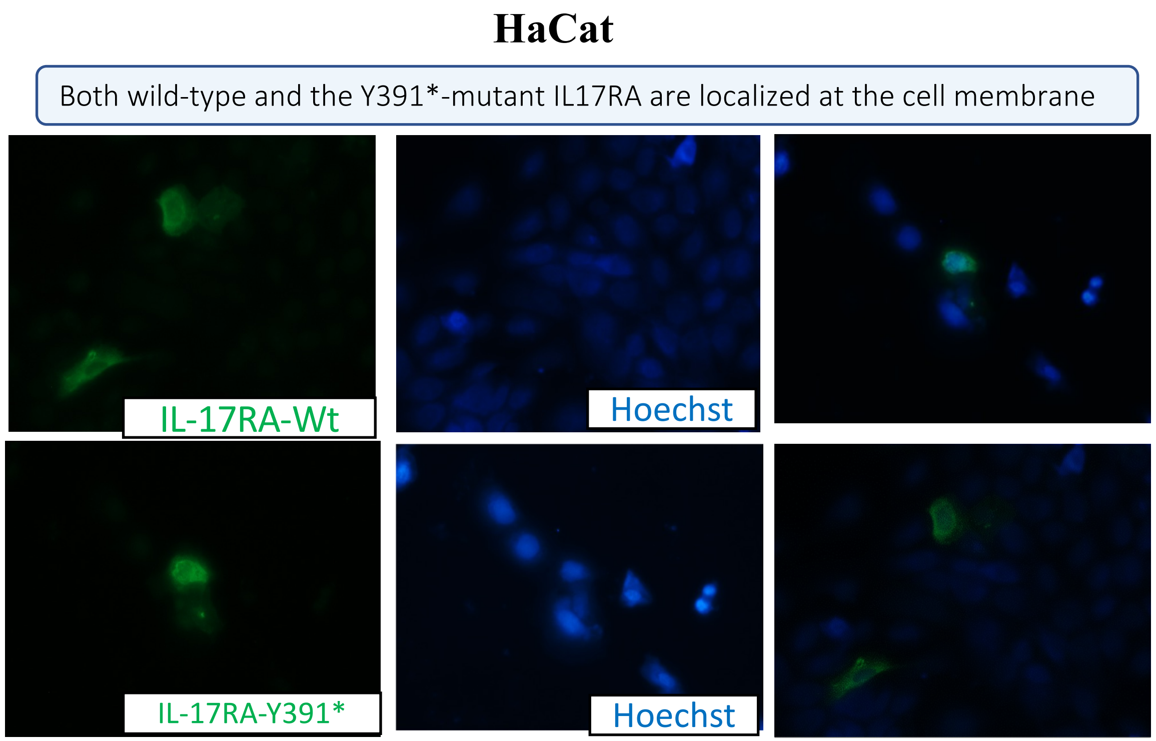

Supplement: Supplementary Figure 1 — Dataflow chart of the variant curation. [file DataSheet1.pdf]
